# Effectiveness of cough etiquette maneuvers in disrupting the chain of transmission of infectious respiratory diseases

**DOI:** 10.1186/1471-2458-13-811

**Published:** 2013-09-08

**Authors:** Gustavo Zayas, Ming C Chiang, Eric Wong, Fred MacDonald, Carlos F Lange, Ambikaipakan Senthilselvan, Malcolm King

**Affiliations:** 1 Mucophysiology Laboratory, Department of Medicine, Faculty of Medicine and Dentistry, University of Alberta, Edmonton, AB, Canada; 2 Department of Medicine, Faculty of Medicine and Dentistry, University of Alberta, Edmonton, AB, Canada; 3 Centre for Lung Health, Northern Lung Function Laboratory, Edmonton General Hospital, Edmonton, Alberta, Canada; 4 Department of Mechanical Engineering, Faculty of Engineering, University of Alberta, Edmonton, Alberta, Canada; 5 Department of Public Health Sciences, School of Public Health, University of Alberta, Edmonton, Alberta, Canada

## Abstract

**Background:**

The effectiveness of recommended measures, such as “cover your mouth when coughing”, in disrupting the chain of transmission of infectious respiratory diseases (IRD) has been questioned. The objective of the current study was to determine the effectiveness of simple primary respiratory hygiene/cough etiquette maneuvers in blocking droplets expelled as aerosol during coughing.

**Method:**

In this study, 31 healthy non-smokers performed cough etiquette maneuvers in an effort to cover their voluntarily elicited best effort coughs in an open bench format. A laser diffraction system was used to obtain accurate, non-invasive, quantitative, real time measurements of the size and number of droplets emitted during the assessed cough etiquette maneuvers.

**Results:**

Recommended cough etiquette maneuvers did not block the release and dispersion of a variety of different diameter droplets to the surrounding environment. Droplets smaller than one-micron size dominate the total number of droplets leaked when practicing assessed maneuvers.

**Conclusions:**

All the assessed cough etiquette maneuvers, performed as recommended, do not block droplets expelled as aerosol when coughing. This aerosol can penetrate profound levels of the respiratory system. Practicing these assessed primary respiratory hygiene/cough etiquette maneuvers would still permit direct, indirect, and/or airborne transmission and spread of IRD, such as influenza and Tuberculosis. All the assessed cough etiquette maneuvers, as recommended, do not fully interrupt the chain of transmission of IRD. This knowledge urges us all to critically review recommended CE and to search for new evidence-based procedures that effectively disrupt the transmission of respiratory pathogens. Interrupting the chain of transmission of IRD will optimize the protection of first responders, paramedics, nurses, and doctors working in triage sites, emergency rooms, intensive care units, and the general public against cough-droplet-spread diseases.

## Study design

Open bench, observational, cough etiquette study.

## Background

Canada was among many countries around the world working in partnership with the World Health Organization (WHO) in preparation for an influenza pandemic outbreak when the severe acute respiratory syndrome (SARS) started to rapidly spread across Asia. SARS is a disease caused by a coronavirus never before seen in humans, the SARS coronavirus (SARS-CoV) [1].

Canada was the country hardest hit outside of Asia, with 438 probable and suspect SARS-CoV cases, including 44 deaths. Canadian health care workers (HCW) endured a high toll during the SARS outbreak while caring for patients. Approximately one out of every four SARS cases affected HCW [2,3].

Health care providers and scientists searched for answers to a number of questions brought up by the SARS-CoV outbreak. The ease of transmission, possibly enhanced by the volume and speed of human migration, and the severity of the disease were both characteristics of great concern. At the time, this outbreak highlighted the inadequacy of national preparedness to detect and respond to emerging infectious diseases, including atypical cases, with neither a curative treatment nor vaccine available to administer. Moreover, the outbreak of the highly pathogenic avian influenza H5N1 virus (2005) brought to the forefront the need to find new, more effective transmission control measures for infectious respiratory diseases (IRD) [4-7]. The most recent example occurred when the world underwent two waves of a moderate pandemic outbreak caused by the influenza A H1N1 virus [8,9].

Vaccination is the main strategy to control outbreaks of epidemic-prone and pandemic-prone infectious respiratory diseases. However, the mutating capacity of most viral pathogens often renders vaccinations ineffective or delay their use until clear identification of the genetic makeup has been made, allowing precious time for the microorganism to spread [10].

Accessing the vaccine against the new strain of influenza virus presented a challenge for countries around the world; in particular, resource-limited countries were extremely concerned that they were left to confront the influenza pandemic largely unprotected.

The WHO, and other agencies, continues to recommend the application of and compliance with basic infection control precautions known as non-pharmaceutical interventions (NPI) as the cornerstone to prevent transmission of droplet-spread epidemic-prone diseases in health care facilities [5,10,11]. Reliance on NPI, such as cough etiquette (CE), demands further inquiries into the efficacy to block cough droplets and to stop the spread of outbreaks provided by these interventions. The term “cough etiquette” has evolved since described by Bone A, *et al.* 2000 [12-19].

A literature search did not yield any scientific or empiric information/evidence regarding the effectiveness of recommended CE maneuvers preventing or blocking the release of bioaerosol droplets, infectious or not, to the surrounding environment. We also found no studies that indicate that CE protects against droplet-spread transmission of IRD.

IRD, including influenza, are transmitted to non-infected subjects when an infected patient expels droplets of different sizes, potentially loaded with pathogens, to the surrounding environment as aerosol when coughing. Cough is a prominent symptom in patients with IRD.

Viral, mycotic, and bacterial IRD gain access to our bodies via the ocular and oral mucosa, and surface mucosa of the upper and lower respiratory system when air is breathed in that carries droplets loaded with pathogens. Many of these pathogens are emerging epidemic-prone (SARS-CoV, avian influenza [AI]) and pandemic-prone (influenza A caused by the H1N1 virus), while others are re-emerging such as *Mycobacterium tuberculosis*.

Gaps still exist in the current cough etiquette knowledge and some intervention strategies are suspected to still be less than optimal. First responders, HCW in emergency departments, lung specialists in Alberta, Canada and very likely in many other countries, continue to voice questions regarding the effectiveness and scientific evidence of CE and other NPI to protect populations and individuals by blocking droplets expelled as aerosol when coughing and preventing outbreaks of IRD [20].

Soon after the SARS and AI outbreaks a new maneuver was added to the definition of respiratory hygiene/cough etiquette: *cover your mouth and nose with your arm, sleeve, or elbow*. We were unable to find who was the first person to publish and describe this maneuver in a peer-reviewed journal, and we did not find any scientific study that supports its implementation.

We found that the Central Maine Medical Center, the Saint Mary’s Regional Medical Center in association with the Maine Medical Association released a video by Dr. Ben Lounsbury (Otorhinolounsburgology [ORL] Productions, 2006) that shows how to cough into your elbow. This seems to be one of the first documented explanations about how to perform the maneuver and the rational to use it [21].

However, this new maneuver is inconsistently recommended in written publications of global health authorities. USA-CDC does not include “cough in your arm/elbow” in its written recommendations, but it appears in the pictorial (poster) recommendation [18].

While no general consensus exists regarding the best description of the respiratory hygiene/cough etiquette among the health agencies mentioned in Table 1, it appears that: “*Cover your mouth and nose with a tissue when you cough or sneeze. Dispose the used tissue in a garbage can. If you don’t have a tissue, cough or sneeze into your elbow or sleeve, not in your hands*” is the most acceptable recommendation.

**Table 1:** Chronological modifications to the definition of CE by national and international health organizations

**Year**	**Organization**	**Cough etiquette developments and important events**
1999	WHO [5,10,11]	Regarding problems with influenza pandemic vaccine production and availability, alternative control measures have to be thought of in advance.
2000	WHO [12]	Cough etiquette: Turning head and covering mouth when coughing, using clothes or spittoons to spit into.
2003		**Severe acute respiratory syndrome**
2005		**Avian influenza**
2006	ECDC [13]	Good respiratory hygiene: covering mouth and nose when coughing or sneezing using tissues and disposing them appropriately.
		NPI is an area neglected by research and those that fund research. There is little evidence and almost no experimental studies to show whether NPI measures work. This topic should receive urgent research attention.
	CPIP^*^[14]	Individuals with respiratory infection should be educated to cover their mouth and nose with a tissue when coughing and dispose of used tissues in waste containers.
	WHO [15]	Recommendations made for cough etiquette have been made more on the basis of plausible effectiveness than controlled studies.
2007	US-CDC [16]	The components of respiratory hygiene/cough etiquette are 1) covering the mouth and nose during coughing and sneezing, 2) using tissues to contain respiratory secretions with prompt disposal into a no-touch receptacle, 3) offering a surgical mask to persons who are coughing to decrease contamination of the surrounding environment, and 4) turning the head away from others and maintaining spatial separation, ideally >3 feet, when coughing.
		Effectiveness of currently recommended infection control measures for individuals is still unknown and additional research is needed to validate NPI and assess their effectiveness.
	CIDAAP^**^[17]	Respiratory hygiene/cough etiquette: Cover the nose/mouth when coughing or sneezing; cough or sneeze into elbow rather than hand.
2009		**Influenza A H1N1 virus pandemic**
	US-CDC [18]	Cover your mouth and nose with a tissue when coughing or sneezing; use the nearest waste receptacle to dispose of the tissue after use, perform hand hygiene.
	ECDC [19]	Cover your mouth and nose using tissues when coughing or sneezing; or cough or sneeze into an arm rather than your hands.
		There have never been trials of respiratory hygiene on either respiratory infections generally, or specifically influenza.
		Most European countries recommended to adopt the simple public health measures of: respiratory hygiene, hand washing, and early self-isolation.
2010	ECDC [19]	Personal protective measures (non-pharmaceutical) for reducing the risk of transmitting human influenza are based in part in evidence from studies and in part on judgment based on public health experience.

*Canadian pandemic influenza plan.

** Committee on infectious diseases of the American Academy of Pediatrics.

Researchers from the Mucophysiology Laboratory at the University of Alberta have been striving to enhance the knowledge on human airways droplet breakup and aerosol emission during coughing. Studying the mucus layer lining the airways under the effect of high-speed cough airflow is essential in determining the cough aerosol composition, droplet breakup, and dispersion. The goal of our research group was to better understand cough bioaerosol composition and to determine the magnitude of droplets emitted by the transmissor and not blocked or controlled while practicing current cough etiquette maneuvers.

Knowledge of the dynamic process of bioaerosol will be used to design efficacious evidence-based prevention interventions against droplet-spread epidemic/pandemic-prone respiratory pathogens. This would reduce the risks of health consequences due to IRD.

This study sought to find an evidence-based response to questions posed by multiple individuals, agencies and organizations dealing with IRD transmission, regarding the effectiveness of recommended NPI/CE to block or control cough droplets to prevent the spread of IRD.

The objective of this study was to assess recommended simple primary prevention measures such as “cover you mouth when coughing” to determine their effectiveness in blocking droplets expelled as aerosol during coughing.

## Methods

### Study design

This was an observational study with a cross-sectional design in which all participants, in an open bench format, were encouraged to practice select recommended cough etiquette maneuvers to cover their voluntarily elicited best effort cough. Although, global health authorities discourage using hands to cover a cough, this maneuver was included to compare its effectiveness with the recommended maneuvers and because many people still use it in many countries.

### Participants

A total of 31 healthy volunteers, ages 18 years and older, were invited and accepted to participate. Participants were recruited through advertised leaflets in public areas around the university campus and none of them declared having asthma, Cystic Fibrosis, or other respiratory conditions. Eligible participants were excluded if they had received expectorants, mucolytics or natural products for respiratory conditions during the previous 30 days, or had developed flu-like symptoms immediately before the study.

### Study site

The study was carried out at the Mucophysiology Laboratory in Heritage Medical Research Centre, University of Alberta, Canada. Environmental conditions at the study site were similar to the indoor conditions found in a hospital reception site with respect to room temperature, humidity and atmospheric pressure.

The University of Alberta Hospital Medical Ethics Committee and the Office of Environmental Health and Safety of the University of Alberta approved the study protocol. Informed consent was obtained from all the participants.

### Study day

The study procedures were explained in detail to all participants by the investigator. Once they had understood the study requirements, all participants were asked to sign an informed consent.

### Measurements

Participants performed a voluntary cough while covering the mouth and nose with the hands, sleeve/arm, tissue, or while wearing a surgical mask. Droplets released or diverted were quantitatively characterized to assess how effective those maneuvers are in controlling the cough aerosol jet. Measurement time per maneuver was 10 seconds.

Every participant was encouraged to voluntarily elicit a “real cough” three times while covering the mouth and nose either with both hands, with a tissue, with the sleeve/arm or while wearing a surgical mask. If during the performance researchers considered that the participant did not make an adequate effort, the participant was asked to repeat the maneuver until an acceptable effort was obtained.

In addition to the acceptable cough efforts, we consistently selected three parameters provided by the laser diffractometer: valid points, skip values and total mass per maneuver. From these parameters we selected which of the three cough efforts was the best. The design implemented in our study was similar to the protocol used when performing a spirometry test: three efforts and select the best effort made. This is a lung mechanics procedure very well established and accepted worldwide.

### Pressure and humidity

Atmospheric pressure sensors (SPC1000, VTI Technologies, Finland) and relative humidity sensors (SHT75, Sensirion, Switzerland) were placed in predetermined areas of potential droplet escape for additional assessment of cough droplets diverted or allowed to flow through barriers.

### Droplet size measurement

For accurate, non-invasive, quantitative measurements in real time of the size and number of droplets as they are emitted during the assessed cough etiquette maneuvers, a laser diffraction system (Spraytec, Malvern, UK) was used. The laser diffraction system has 60 size bins with the capability of measuring the concentration of droplet sizes from 0.1 micron (μm) to 900 μm every 0.4 millisecond.

The Spraytec He-Ne (Helium-Neon) laser diffractometer is composed of transmitter and receiver modules. Expelled respiratory aerosols pass through a cylindrical measurement zone with a volume of 7.85 cm^3^ through a path of 100 mm length and 10 mm diameter. The path length is estimated as the distance through the spray plume that the laser beam travels. As the droplets pass through the laser measurement volume zone, laser light from the transmitter is scattered by the respiratory aerosol producing light diffraction patterns, which are measured by optical detectors on the receiver modules. The light signals are then converted into electrical signals to process a droplet size distribution, under the assumption that each droplet is a perfect sphere. The angle at which a droplet diffracts light is inversely proportional to its size.

The He-Ne laser diffractometer was set to measure the droplet concentration of a single cough event crossing the measurement zone every 0.4 milliseconds (2.5 GHz) during a manually triggered time of 10 seconds. Units of droplet concentration are expressed as average rate of # of droplets/cm^3^/second, averaged from the beginning to the end of the cough.

**Figure 1:**
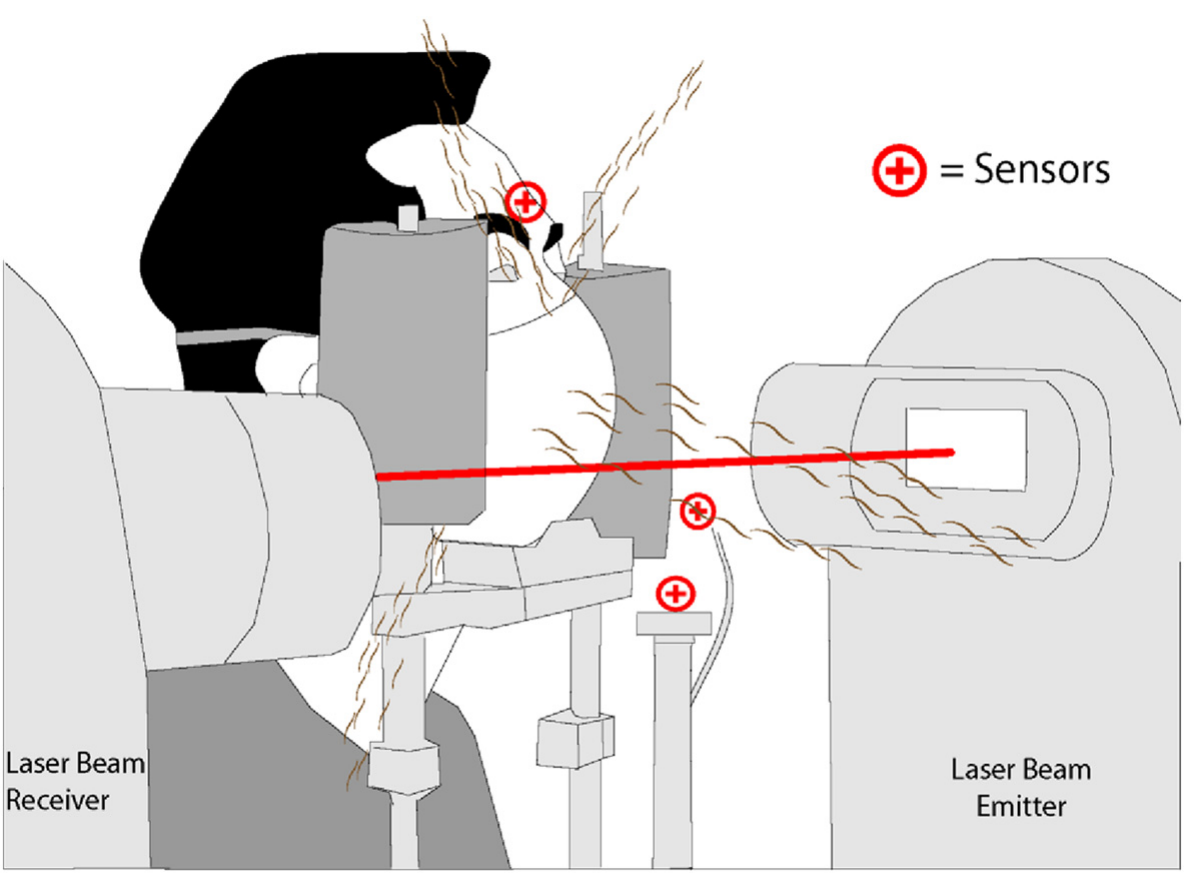
Laser and sensor arrangement for assessment of surgical mask and hand as barriers.

**Figure 2:**
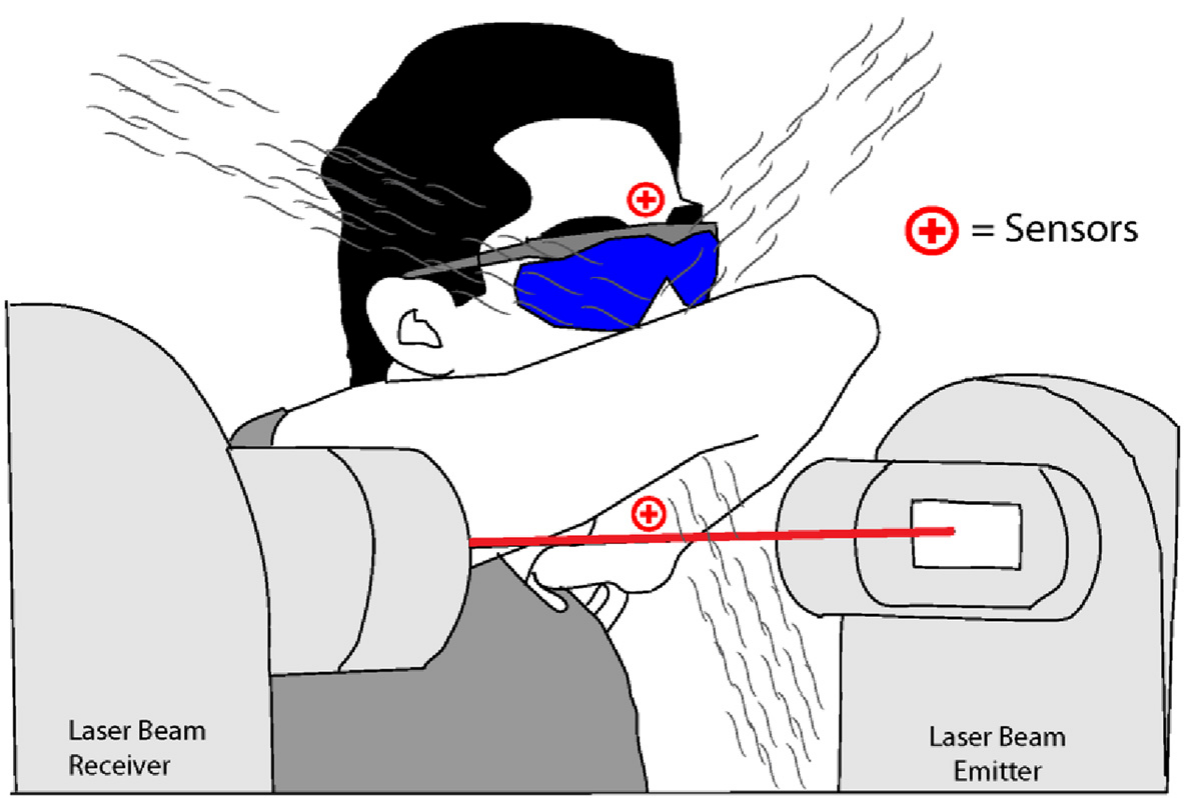
Laser and sensor arrangement for assessment of using the arm/sleeve as a barrier.

**Figure 3:**
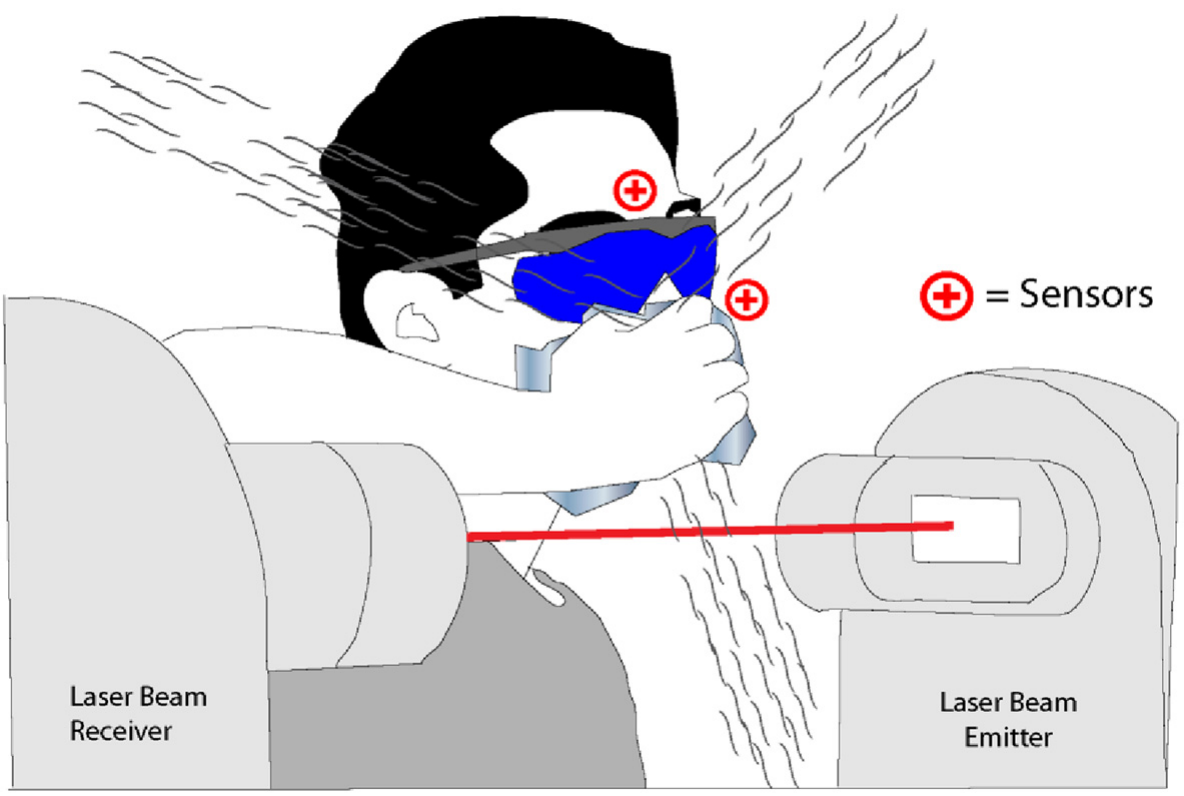
Laser and sensor arrangement for assessment of using a tissue as a barrier.

For the present study, the laser beam was directed from left to right at 17 cm in front of the face of the participant when covering mouth/nose using a surgical mask or using both hands (Figure 1), or at 5 cm below the chin when covering mouth/nose using the sleeve/arm or using a tissue (Figures 2 and 3). As the figures indicate, droplets are also diverted in other directions not captured in our measurement. Because of this, our measurements can be used to indicate if there is or not emission of droplets, but they cannot be used to assess the total amount of droplets emitted. Since there were no precedents regarding the use of a laser diffractometer in an open bench format to assess cough etiquette and NPI maneuvers, the 17 cm and 5 cm distances were selected by the researchers. This decision was based mainly on the grounds of safety: the distance and positioning of the laser beam was selected to reduce potential contact with the eye or face of the participants. This would maximize the detection of cough airflow droplets as they were expelled, assuring in a single, complete, and uninterrupted event that they would cross the centre of the measurement zone without any interference to the flow of the aerosol. An open fume hood facing the participants removed airborne dust particles and airborne cough droplets from the environment. We did not measure evaporation rate. Deposition losses were not a factor in the open bench design.

To perform the maneuver of “cough in your elbow, arm, sleeve” the forearm is flexed against the arm and placed in front of the mouth. Preliminary measurements with pressure and humidity sensors indicated that the cough airflow is diverted mostly below the elbow.

### Statistical analysis

The data were expressed as mean ± standard deviation (SD) unless otherwise stated. No statistical comparison was carried out in this observational study.

### Research procedures

#### Assessing surgical mask and hands

Participants were instructed to place their face in a modified device similar to the head brace used by optometrists. This device prevented participants from interfering with the path of the laser beam.

Four (4) sensors were placed in areas of concern around the face of the participant: one humidity sensor was placed close to the nose bridge above the surgical mask or the hands; two (2) humidity sensors were mounted on a post which stood approximately 15 cm in front of the participant mouth, one about 10 degrees angle below and the second about 30 degrees angle below in a parallel position; and one pressure sensor was mounted on the aforementioned post at approximately 30 degrees below in a parallel position (Figure 1).

#### Sleeve/arm and tissue

Participants were asked to use only the right arm for practical purposes and were instructed to wear laser He/Ne safety eyewear (Sperian, RI, USA). This eyewear complies with ANSI Z136.1 standards.

Three (3) sensors were placed in areas of concern around the face of the participant: two (2) sensors, one pressure and one humidity, were placed close to the nose bridge above the flexed elbow or above the right hand holding the tissue; and one humidity sensor was placed either in the ventral part of the right forearm about 7–10 cm from the wrist or next to the left cheek when using a tissue (Figure 2).

In this study we will emphasize results related to covering the mouth/nose using the sleeve and briefly comment about the other public health maneuvers.

## Results

During the period of assessment (March – May, 2010) we detected the following averages (± standard deviation) inside the testing site: atmospheric pressure = 91.8 ± 1.1 kPa, relative humidity =19.0 ±3.9% RH, and temperature = 22.7 ± 2.0°C.

Cough droplets diverted or dispersed to the surrounding environment while performing cough etiquette maneuvers were assessed in 19 male and 12 female participants. All participants self-identified as non-smokers, with the exception of one male who declared he was a long-term (30+ years) ex-smoker.

Data acquired with pressure and humidity sensors while performing the procedure of CE is shown in the Tables 2 and 3. The pressure values are average delta pressure fluctuations and the humidity values are average delta increments over ambient.

**Table 2:** Data from pressure and humidity sensors of cough etiquette: using sleeve/arm and tissue

**Maneuver**	**Sleeve/arm**			**Tissue**		
**Number of subjects**	**25**	**24**	**25**	**25**	**24**	**25**
Type	Pressure (kPa)	Relative humidity (%)		Pressure (kPa)	Relative humidity (%)	
Location	Nose bridge	Nose bridge	Wrist	Nose bridge	Nose bridge	Left cheek
Variation	0.4	7.13	15.13	0.5	8.95	15.44

Sensors placed around the barrier detect cough airflow redirected by the maneuvers. Values in this table are above the average room value detected during the experiment.

**Table 3:** Data from pressure and humidity sensors of cough etiquette: using hands and surgical mask

**Maneuver**	**Hands**				**Surgical mask**			
**Number of subjects**	**24**	**24**	**24**	**21**	**24**	**24**	**23**	**24**
Type	Pressure (kPa)	Relative humidity (%)			Pressure (kPa)	Relative humidity (%)		
Location	Post	Post	Above post	Nose bridge	Post	Post	Above post	Nose bridge
Measure	1.37	35.9	39.6	24.7	0.10	5.5	22.5	12.1

Sensors placed around the barrier detect cough airflow redirected by the maneuvers. Values in this table are above the average room value detected during the experiment.

The large number of droplets of different size, generated by the best-effort cough and detected by the laser diffractometer, were normalized and expressed as the average rate of *number of droplets per cubic centimeter per second*. These averages were grouped into six (6) categories according to droplet size: a) < 0.5 μm, b) 0.5 to 1 μm, c) >1.0 to 2.5 μm, d) >2.5 to 10 μm, e) >10 to 100 μm and f) >100 μm. The results per category, tabulated and summarized in Table 4, were compared with their respective control value. Control data of an open bench cough aerosol obtained from 44 participants was presented in an article published by Zayas *et al.*[22].

**Table 4:** Average rate of droplets detected during respiratory hygiene/cough etiquette maneuvers in 31 participants

**Size/maneuver**	**Sleeve**	**Tissue**	**Hand**	**Surgical mask**	**Control n = 44**
**N** <0.5 μm	4.13E + 07	6.22E + 07	5.13E + 07	4.40E + 07	1.99E + 07
0.5 μm < **N** <1.0 μm	5.36E + 05	5.78E + 05	5.29E + 05	3.40E + 05	3.58E + 05
1.0 μm < **N** <2.5 μm	7.76E + 04	8.47E + 04	5.50E + 04	5.05E + 04	4.16E + 04
2.5 μm < **N** <10 μm	8.52E + 04	1.12E + 05	6.64E + 04	6.53E + 04	4.18E + 04
10 μm < **N** < 100 μm	4.98E + 03	6.10E + 03	4.66E + 03	6.12E + 03	2.64E + 03
**N** >100 μm	0	0	0	0	0

Unit: # droplets/cc/second.

Control: Size and number of droplets expelled by healthy non-smokers when coughing. Data acquired from an expanding unobstructed cough aerosol.

CE data: The short distance from the mouth to the barrier prevents the expansion of the cough plume, and the shape of the barrier redirects a more concentrated flow across the measurement zone. The non-expanding concentrated plume would bring droplets travelling in the periphery closer to the center of the plume increasing the number of droplets accounted for.

Particles deposited within the fiber network of tissues and surgical masks during the manufacturing process might be dislodged when coughing, hence increasing the number of items detected by the system.

The average volumetric mean diameter and standard deviation of the droplets expelled as aerosol when coughing that crossed the measurement volume zone per CE maneuver is as follows: sleeve 0.31 ± 0.06 *μm*, tissue 0.30 ± 0.02 μm, hands 0.30 ± 0.04 μm, and surgical mask 0.30 ± 0.03 μm. The standard deviation in the size distribution is of the average volumetric mean diameter.

## Discussion

While global health authorities and agencies do not recommend covering the mouth/nose using bare hands when coughing, this procedure was included in our study for comparison of droplets released into the environment when using hands. We fully agree that when using the hands to cover the cough, respiratory pathogens could be transmitted to other individuals if contact precautions are not followed.

Major findings in this study include: a) recommended respiratory hygiene/cough etiquette maneuvers do not block or contain cough droplets expelled as aerosol from dispersing towards the surrounding environment. b) Droplets smaller than one-micron size dominate the total number of droplets released when practicing cough etiquette. c) All the assessed cough etiquette maneuvers have the potential to permit direct, indirect and/or airborne transmission of respiratory infections. d) Data acquired in this study support the conclusions that all recommended respiratory hygiene/cough etiquette allow the spread of epidemic-prone IRD outbreaks, instill a false sense of security, and merit a critical review.

This study was implemented to close the gaps in knowledge that exist regarding how successful recommended NPI are in blocking or controlling coughs droplets. During the expulsive phase of coughing airflow comes from inside the chest to the external environment at approximately up to 100 km/h [23,24]. Droplets coming out of the mouth of a person that coughs will very likely be a mixture of droplets of different sizes generated in different levels of the respiratory systems.

In Table 1 we have summarized the chronological development and recommendation of respiratory hygiene and cough etiquette maneuvers since its inception, issued by national and multinational health agencies (WHO, US-CDC, Health Canada, European-CDC). The summary shows that after the SARS and avian influenza outbreaks all agencies increased the frequency and emphasis on the use of NPI/CE measures to control the spread of IRD.

Cough droplets are centerpiece in the chain of transmission of IRD. During the transmission process, infected individuals expels numerous droplets of different sizes into the air every time they cough. Infectious respiratory pathogens whether virus, bacteria or fungus, are dispersed towards the outside environment when droplets formed in the mucus layer lining the airways of an infected patient are exposed to the high-speed cough airflow.

Zayas *et al.* found in a previous cough aerosol open bench study [22] that, per cough, healthy non-smokers expelled millions of droplets of different sizes as aerosol. Results from that study indicated that droplets smaller than 10 μm constitute the largest majority. Droplets of such size are able to penetrate deep into the respiratory system.

We can also conclude that due to the size of the droplets moving at high-speed from inside of the respiratory system and arriving to an external environment with different level of humidity and temperature, evaporation might occur rapidly. We can also expect that the airborne route is the dominant route in IRD transmission, independent of pathogen, due to the millions of rapidly evaporating and nuclei forming droplets.

When the high-speed cough airflow coming out of the respiratory system encounters a physical barrier, the flow either goes through the barrier or alongside it towards the areas that present the lowest resistance. Therefore, under the light of this data we conclude that assessed recommended respiratory hygiene/cough etiquette maneuvers are unable to stop or prevent the escape of all inhalable droplets contained in the cough bioaerosol. This implies that in the case of a person infected with an epidemiological important pathogen, recommended NPI/CE will still permit the dispersion of numerous infectious droplets, increasing the risk of exposure, infection of susceptible individuals.

**Figure 4:**
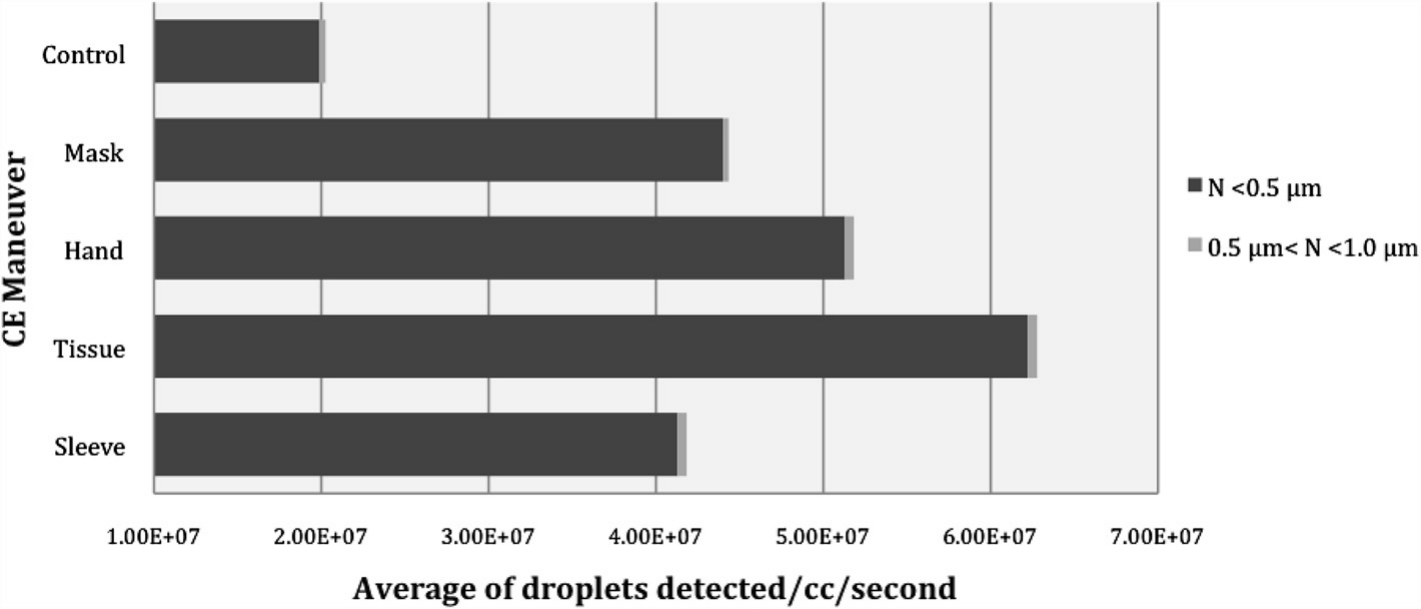
**Average droplets detected per cough etiquette maneuver.** Control: Size and number of droplets expelled by healthy non-smokers when coughing. Data acquired from an expanding unobstructed cough aerosol. CE data: The short distance from the mouth to the barrier prevents the expansion of the cough plume, and the shape of the barrier redirects a more concentrated flow across the measurement zone. The non-expanding concentrated plume would bring droplets travelling in the periphery closer to the center of the plume increasing the number of droplets accounted for. Particles deposited within the fiber network of tissues and surgical masks during the manufacturing process might be dislodged when coughing, hence increasing the number of items detected by the system.

Data from Figure 4 indicate that while practicing assessed CE maneuvers the laser diffraction system detected a larger number of droplets compared to our control group, which was an *unobstructed open bench cough*. This increase in droplet numbers should not be used to infer an increased total emitted amount, because the exact relationship between the emitted volume and the measured volume is not known. In a previous article [22] we considered that the distance the cough airflow travels before crossing the measurement zone might prevent droplets travelling in the periphery of the expanding plume be accounted for. Another factor for this increase would be the shape of the barrier in front of the cough airflow.

The short distance from the opening of the mouth to the inner surface of the barrier and the barrier in front of the cough both prevents the conic expansion of the cough aerosol when exiting the mouth. When a person covers the cough with the bare hands or with a tissue, they tend to close all their fingers tightly against each other, and press closely each hand against the other by the cubital border of the palm.

Such position of the hands forms a vertically elongated concave pear-shape space with the tips of the middle and index fingers pressing against both sides of the nose bridge, and placing the radial side of the index fingers alongside the nose and thumbs pressing firmly against the cheeks and side of the mouth. Placing the hands and fingers as described form a sort of impermeable seal leaving a separation between the wrists and below the chin that creates an open area of low or no resistance, redirecting a more concentrated flow downward. The redirected non-expanding plume exiting the hand barrier would lead to a larger number of droplets accounted for when a concentrated plume crosses the measurement zone.

A similar situation occurs when the cough airflow strikes the sleeve of a folded arm, albeit leaving more low resistance areas. Additionally, the distance that the plume diverted by the barrier has to travel to cross the measurement zone is shorter, about five centimeters.

This is our initial explanation, yet there may be others. Coughing into the tissue might also dislodge particles deposited within the fiber network during the manufacturing process, increasing number of items detected by the system. In any case, the fact that we measure droplet concentrations of the same order of magnitude as in the control case fully supports our major findings.

We agree that practicing any of the recommended maneuvers when coughing may be acceptable during a seasonal influenza. However, when facing a deadly and severe droplet-spread, epidemic/pandemic-prone outbreak, health authorities must consider procedures with higher effectiveness. Nicoll [13] suggests that personal (non-pharmaceutical) protective measures should receive urgent attention from researchers and from those funding research.

A deeper insight into mucus-pathogen interaction and airways droplet breakup, as well as dispersion and bioaerosol control, is an essential component in assisting lung health researchers, mathematicians, computer modelers, epidemiologists, policymakers, public health workers, and the entire health care system in rich and poor countries alike, in the design of sound, evidence-based IRD dispersion, transmission control and preventative measures. It will also further advance policies and products to optimize protection against transmission of epidemic-prone droplet-spread respiratory diseases.

It is difficult to critically appraise the results acquired in this study with respect to other published studies assessing CE maneuvers, including facemasks, for a number of reasons. Most of them used closed systems with different designs, equipment with much lower resolution, and biased droplet collection to characterize the size and number of droplets. This differs from our design: an open cough, real time measurement in humans. Those that studied cough aerosol generated by machines based their results on samples taken from a simulated cough plume. Those who used human volunteers to assess facemasks, like Milton *et al.*[25], took non-real time measurements from samples of cough aerosol acquired during a period of 30 minutes with and without the mask.

However, a cluster randomized trial conducted in France by Canini *et al.*[26] (PLoS ONE, 2010), assessing the effectiveness of facemasks for limiting influenza transmission in households, was prematurely interrupted after the control arm and the case arm failed to show the effectiveness of facemasks. Another study in Germany, conducted by Suess *et al.*[27] (BMC Infectious Diseases, 2012), showed that household transmission of influenza can be reduced when using facemasks plus intensified hand hygiene, not when wearing facemasks alone.

We reiterate that our study was implemented to close the existing gaps in knowledge regarding the effectiveness of recommended NPI in blocking transmission of IRD or in controlling coughs droplets coming from inside the chest to the external environment. Although numerous published articles claim that surgical masks block cough droplets, the critical question is: do surgical masks stop IRD transmission?

France and Germany have studies showing that surgical masks are not effective in blocking transmission of viral diseases or need a lot of help from vigorous hand washing. The USACDC stated very recently that *"facemasks may be effective in blocking splashes and large-particle droplets,… a facemask by design, does not filter or block very small droplets"*[28].

Our previous study [22] shows that the majority of droplets released when coughing are smaller than 10 microns in size. Our current study shows that droplets of similar magnitude are still released while using surgical masks. Therefore the French and German studies mentioned above support our conclusion that all recommended respiratory hygiene/cough etiquette, including facemasks, allow the transmission of epidemic-prone IRD outbreaks due to the nature, size, and number of cough droplets.

The new knowledge acquired in this study would provide the scientific support needed to design evidence-based preventative measures and alternatives in existing technologies to optimize public health practices in bioaerosol control. Such knowledge might suggest and lead to new avenues to mitigate the effects of the droplet-spread outbreak in protecting health care workers, the general public and institutions.

Despite the inconsistencies among global health authorities and their cough etiquette recommendations, the indication of “cover your cough with your elbow/arm/sleeve” has reached a phenomenal, close to universal acceptance, including elementary school children who are being successfully trained to practice it. Furthermore, this remarkable compliance around the globe has occurred in a very short period of time. Society has adopted this maneuver without asking for or demanding scientific evidence.

The prompt acceptance, implementation, societal/individual behavior modification, and global dissemination of the maneuver present the scientific community with a dilemma: why was this maneuver so popular in light of the lack of evidence to support it? Involved are several facts and actions: a) no peer review publication documenting this maneuver, b) no scientific evidence supporting the effectiveness of such a maneuver, and c) no scientist author, developer or designer fathering such a maneuver.

Lounsbury [21] presented this maneuver in a humoristic and entertaining video format. The video was a huge success in terms of public acceptance and secured the support of various medical and community associations. Soon after the release of the video the world was witnessing people of all ages, including kindergarten aged children, practicing the maneuver.

The lack of scientific evidence supporting this particular maneuver is a valid argument for most global health agencies to avoid including it in their set of written recommendations, however it is still conditionally included in pictorial recommendations, as seen in CDC campaigns [18].

Whether or not this particular maneuver is based on scientific evidence, the general public accepted it and voluntarily adopted a change in behavior. This can serve as an example for the scientific community to understand how knowledge should be structured, translated, and communicated to get the message across and subtly induce positive behavioral change in the population.

In summary, assessed public health maneuvers, including facemasks, do not fully protect against the millions of smaller cough droplets, as micron size droplets dehydrate rapidly, form nuclei, remain airborne, and penetrate deep in the lung when inhaled, augmenting the risk of infection, of developing disease, and even increasing mortality due to transmitted infection.

## Conclusions

Researchers at the Mucophysiology Research Centre, University of Alberta have characterized in an open bench format the cough aerosol and determined the size and, more importantly, the number of droplets expelled when coughing [22]. This achieves a critical step that could contribute to enhancing control of IRD, like influenza A caused by the H1N1 virus.

The results acquired in this study indicate that all CE maneuvers assessed do not block droplets expelled as aerosol when coughing. This aerosol can penetrate profound levels of the respiratory system. Practicing these assessed primary respiratory hygiene/cough etiquette maneuvers would still permit direct, indirect, and/or airborne transmission and spread of IRD, such as influenza and Tuberculosis.

Acquired data suggests that in the case of an individual infected with a highly pathogenic microorganism, infectious cough droplets would still be released to the surrounding environment when covering the mouth/nose with any of the assessed respiratory maneuvers, allowing the probability of infecting susceptible individuals.

Although it remains possible and even logical that transmission is reduced somewhat (some droplets must be captured during those maneuvers), this study was not designed to carry out rigorous measurements of this reduction. Measurement from our study established that all maneuvers, as recommended, are equivalently inadequate at completely blocking cough-droplets, even though we cannot say how much in terms of percentage reduction.

Furthermore, our results coincide with the assertion previously stated by researchers and global public health authorities, confirming that the assessed non-pharmacological interventions used during and after the two recent waves of the pandemic caused by the influenza A H1N1 virus were based on a very fragile scientific base.

To control the epidemic spread of airborne diseases, the path from the infected person (transmissor) to a non-infected person (recipient) must be effectively interrupted. This study presents us with a serious challenge: the need to search for new procedures that effectively block cough bioaerosol to interrupt the chain of transmission and spread of IRD.

Achieving such a challenge would optimize the protection of first responders, paramedics, nurses, and doctors working in triage sites, emergency rooms, intensive care units, and the general public.

Therefore we all must strive to design highly effective maneuvers and/or devices to block cough droplets of all sizes from dispersing into the surrounding environment, enhancing the control of transmission of IRD and optimizing protection of all members of our society. We must also strive to understand how knowledge should be structured, translated, and communicated to get the message across and subtly induce positive behavioral change in the population.

## Competing interests

The authors declare that they have no competing interests.

## Authors’ contribution

JGZ and MK developed the concept of cough etiquette assessment, designed the cough etiquette study, interpreted the data and drafted the manuscript. EW and FM performed clinical assessment of participants, contributed to develop the basis for the cough etiquette and with clinical interpretation and review of the manuscript. CL contributed to strengthen the methodology of the study and determine the best data acquisition device and revision of the manuscript. AS contributed with analysis of data and with critical revision and interpretation of the manuscript. MCC made key contributions by making the laser device to operate according to the study design, with acquisition of data, and by developing the software to optimize data acquisition and the database. All authors read and approved the final manuscript.

## Pre-publication history

The pre-publication history for this paper can be accessed here:

http://www.biomedcentral.com/1471-2458/13/811/prepub
